# Increased ApoE Expression in Follicular Fluid and the ApoE Genotype Are Associated With Endometriosis in Chinese Women

**DOI:** 10.3389/fendo.2021.779183

**Published:** 2021-11-10

**Authors:** Ya-Jing Liu, Fen Xing, Kai Zong, Meng-Yao Wang, Dong-Mei Ji, Yu-Hang Zhao, Yun-He Xia, An Wang, Ling-Ge Shi, Si-Min Ding, Zhao-Lian Wei, Jin-Ping Qiao, Xin Du, Yun-Xia Cao

**Affiliations:** ^1^ Reproductive Medicine Center, Department of Obstetrics and Gynecology, The First Affiliated Hospital of Anhui Medical University, Hefei, China; ^2^ National Health Commission (NHC) Key Laboratory of Study on Abnormal Gametes and Reproductive Tract, Anhui Medical University, Hefei, China; ^3^ Key Laboratory of Population Health Across Life Cycle (Anhui Medical University), Ministry of Education of the People’s Republic of China, Hefei, China; ^4^ Anhui Province Key Laboratory of Reproductive Health and Genetics, Anhui Medical University, Hefei, China; ^5^ Biopreservation and Artificial Organs, Anhui Provincial Engineering Research Center, Anhui Medical University, Hefei, China; ^6^ Technical Center of Hefei Customs District, Hefei, China; ^7^ First Clinical Medical College, Anhui Medical University, Hefei, China; ^8^ Department of Clinical Laboratory, The First Affiliated Hospital of Anhui Medical University, Hefei, China; ^9^ 901st Hospital of People’s Liberation Army (PLA) Joint Logistic Support Force, Hefei, China

**Keywords:** apolipoprotein E, endometriosis, follicular fluid, genotype, multifactor prediction model

## Abstract

More than 10% of women suffer from endometriosis (EMT) during their reproductive years. EMT can cause pain and infertility and requires further study from multiple perspectives. Previous reports have indicated that an increase inapolipoprotein E (ApoE) may be associated with a lower number of retrieved mature oocytes in older women, and an association between ApoE and spontaneous pregnancy loss may exist in patients with EMT. The purpose of this study was to investigate the existence of an increase in ApoE in follicular fluid (FF) and the possible relationship between ApoE and EMT in Chinese women. In the current study, 217 Chinese women (111 control subjects and 106 EMT patients) were included. The ApoE genotypes were identified by Sanger sequencing. We found that ApoE expression in FF was higher in patients with EMT than in the control group. In addition, a significant difference in ApoE4 carriers (ϵ3/ϵ4, ϵ4/ϵ4) was found between the control subjects and the patients with EMT. Furthermore, a nonparametric test revealed significant differences in the numbers of blastocysts and high-quality blastocysts, but not the hormone levels of FSH, LH, and E2, between the two groups. We also established a multifactor (BMI, high-quality blastocysts, and ϵ4) prediction model with good sensitivity for identifying patients who may suffer from EMT. Our results demonstrate that ApoE expression in FF is increased in EMT, the ApoE-ϵ4 allele is significantly linked to EMT, and a combined analysis of three factors (BMI, high-quality blastocysts, and ϵ4) could be used as a predictor of EMT.

## Introduction

Endometriosis (EMT), which is a common disease in women of reproductive age (1), is characterized by a chronic inflammatory process (2, 3) in which endometrial tissue grows outside the uterine body. EMT affects more than 10% of women of reproductive age in the world, and approximately 30%–50% of these women experience chronic pain and infertility (4). The specific etiology of EMT has not yet been clarified, and its incidence may be associated with a countercurrent flow of menstrual blood, sex hormone disorders, immune factors, and genetic factors (5). Thus, an investigation of the factors contributing to EMT might be crucial to elucidating its etiology.

An increasing number of studies indicate that apolipoprotein E (ApoE) is linked to inflammation and disease risk, and a higher proinflammatory state is associated with the ϵ4 allele (6). ApoE, which is a multifunctional protein that is widely found in mammals, consists of 299 amino acids and has a molecular mass of 34 kDa (7). ApoE is a component of very low-density lipoprotein, which transports peripheral cholesterol to the liver for metabolism. Thus, the important role of ApoE in lipid transport has been widely studied (8, 9). The ApoE genotype also affects the cholesterol and lipid levels in plasma and brain (10). The human ApoE gene is encoded by the long arm of chromosome 19. ApoE is primarily synthesized in liver and brain tissues and is also expressed in the adrenal gland, ovary, kidney, and skeletal muscle (11). In addition, both rat and human ovarian granulosa cells produce ApoE *in vitro*. Polymorphisms in ApoE were first proposed by Utermann (12). The three most common isoforms of ApoE are ApoE2, ApoE3, and ApoE4, and these combine to form six genotypes: ϵ2/ϵ2, ϵ3/ϵ3, ϵ4/ϵ4, ϵ2/ϵ3, ϵ2/ϵ4, and ϵ3/ϵ4. The differences between these genotypes are mainly due to differences in the amino acids at positions 112 and 158 in the amino acid chain of the protein. ApoE3 contains a cysteine residue at position 112 and an arginine residue at position 158 (E3=Cys112/Arg158), whereas ApoE2 has cysteine residues at both positions (E2=Cys112/Cys158), and ApoE4 contains arginine residues at both sites (E4=Arg112/Arg158) (13). ApoE-ϵ4 is a risk factor for a variety of diseases, such as Alzheimer’s disease (AD) (14), cardiovascular disease (15), and hyperlipidemia (16). ApoE is a major source of cholesterol precursors for the synthesis of ovarian estrogen and progesterone (13), and a relationship between the serum estrogen level and ApoE exists in human ovarian follicular fluid (FF) (17), which suggests that ApoE might play an important role in the ovary. Furthermore, a previous study indicated that an increase in the ApoE levels might be linked to a lower number of retrieved mature oocytes in older women (18), and an association between ApoE and spontaneous pregnancy loss may exist in patients with EMT (19). This study aimed to investigate whether ApoE expression is increased in FF and explore the possible association between APOE genotypes and EMT in Chinese women.

## Materials and Methods

### Subjects

The control group consisted of subjects undergoing *in vitro* fertilization and embryo transfer (IVF-ET) for tubal factors and/or male factor infertility in the Reproductive Medicine Center, the First Affiliated Hospital of Anhui Medical University. The experimental group consisted of patients diagnosed with EMT at the First Affiliated Hospital of Anhui Medical University. A total of 111 controls and 106 patients with EMT were enrolled in this study. The majority of the patients with EMT included in the study were diagnosed with ovarian chocolate cysts, and the rest of the patients with EMT were diagnosed laparoscopically. The inclusion criteria were as follows: (1) patients who met the clinical diagnostic criteria for EMT (20); (2) patients aged 21–36 years; and (3) patients who voluntarily participated in the study and signed informed consent forms. The exclusion criteria were as follows: (1) patients with malignant tumors; (2) patients with metabolic diseases; and (3) patients with serious endocrine diseases. This study was approved by the Medical Ethics Committee of the First Affiliated Hospital of Anhui Medical University. Each subject provided written informed consent prior to entering the study.

### Determination of the ApoE Genotype

DNA was extracted from peripheral blood samples. Primers were designed, and PCR amplification was performed. Sanger sequencing was used to determine the APOE genotype.

### Follicular Fluid Collection

Follicular fluid (FF) was collected from women undergoing *in vitro* fertilization and embryo transfer (IVF-ET) after obtaining informed patient consent and approval from the ethical committees at the First Affiliated Hospital of Anhui Medical University. Briefly, after retrieval of the cumulus-oocyte complex, the discarded FF was centrifuged for 10 min at 12,000 rpm and 4°C. The supernatant was collected and maintained at −80°C until assayed.

### Western Blot

Protein was extracted from FF according to a previously reported procedure. The denatured protein was separated by 12% SDS-PAGE and transferred to a PVDF membrane. Then, the membranes were blocked with 5% nonfat dry milk diluted with Tris-buffered saline with Tween 20 (TBST) for 2 h and incubated with antihuman ApoE antibody (1:5,000) and tubulin (Sigma) (1:10,000) overnight at 4°C. The next day, the membranes were washed three times in TBST and incubated with horseradish peroxidase (HRP)-conjugated rabbit antigoat or goat antirabbit secondary antibodies for 1.5 h at room temperature. The protein bands were detected using an enhanced chemiluminescence detection system (Bio-Rad, Hercules, CA, USA).

### Data Analysis

The average values ± standard deviations were used to describe the general characteristics of the control group and patients with EMT, and the APOE alleles (ϵ2, ϵ3, and ϵ4) were examined against Hardy-Weinberg equilibrium by *χ* ^2^ test. The counting data were tested by *t*-test, nonparametric test, or *χ* ^2^ test, and all the data were analyzed using SPSS 23 software. *p* < 0.05 was considered to indicate statistical significance. Univariate analysis was performed to identify the variables with significant differences between the two groups. Multivariate logistic regression analysis was conducted to construct a histogram model for predicting endometriosis using R4.0.3.

## Results

### ApoE Levels in the FF of the Control Group and Patients With EMT

**Figure 1:**
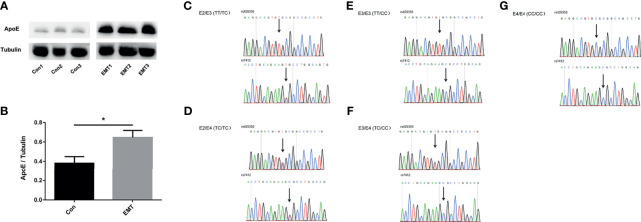
ApoE levels in the follicular fluid (FF) of the control group and patients with EMT. **(A, B)** Representative Western blotting results showing ApoE expression in the FF of the control subjects and the patients with EMT. Tubulin was used as an internal control. The data are expressed as the means ± SEMs; *p < 0.05. **(C–G)** Representative Sanger sequencing for E2/E3,E2/E4,E3/E3, E3/E4,E4/E4 genotype; The arrows indicate the positionof the single nucleotide polymorphism.

The follicular microenvironment in patients with EMT is closely related to infertility; thus, ApoE expression was detected in the FF of patients with EMT. As shown in **Figures 1A, B**, ApoE expression was higher in the FF of patients in the EMT group than in that of the control subjects (*p* < 0.05). ApoE genotypes were confirmed by Sanger sequencing (**Figures 1C–G**).

### Descriptive Characteristics of the Control and EMT Groups

The general characteristics of the control group and the patients with EMT are shown in **Table 1**. The control group included 111 subjects between 23 and 34 years of age, and the EMT group included 106 patients between 21 and 36 years of age. No significant differences in age (*p* = 0.055), infertility time (*p* = 0.065), or egg number retrieved (*p* = 0.091) were detected between the groups, but significant differences in the BMI (*p* = 0.024), number of blastocysts (*p* = 0.018), and number of high-quality blastocysts (*p* = 0.004) were found.

**Table 1:** General characteristics of control subjects and patients with EMT.

Parameters	Control (*n* = 111)	EMT (*n* = 106)	*t*/*z*	*p*-value
Age	28.369 ± 2.347	29.094 ± 3.109	−1.932	0.055
BMI (kg/m^2^)	22.661 ± 3.968	21.626 ± 2.636	2.275	0.024^*^
Infertility time	3 (2, 4)	2 (1, 4)	−1.846	0.065
Numbers of eggs	12 (9, 20)	11 (7, 17)	−1.688	0.091
Numbers of blastocysts	5 (3, 8)	3.5 (2, 6)	−2.369	0.018^*^
High-quality blastocysts	5 (2, 7)	3 (1, 5)	−2.888	0.004^**^

The values show the means ± SD or medians. Student’s t-test and χ^2^ were performed. BMI, body mass index. Six individuals were included in the statistical analysis. ^*^p < 0.05; ^**^p < 0.01.

The frequencies of APOE genotypes in the control and EMT groups were as follows: control group, 0 for ϵ2/ϵ2, 1.8% for ϵ2/ϵ3, 0 for ϵ2/ϵ4, 92.8% for ϵ3/ϵ3, 4.5% for ϵ3/ϵ4, and 0.9% for ϵ4/ϵ4; patients with EMT, 0 for ϵ2/ϵ2, 13.2% for ϵ2/ϵ3, 1.9% for ϵ2/ϵ4, 68% for ϵ3/ϵ3, 13.2% for ϵ3/ϵ4, and 3.7% for ϵ4/ϵ4 (**Table 2**). The distribution of ApoE genotypes in both the control and EMT groups followed Hardy-Weinberg equilibrium (**Table 2**).

**Table 2:** Frequencies of the ApoE genotype and frequencies in the control group and patients with EMT.

	Control (%)	EMT (%)	*p*-value
Genotype			
ϵ2/ϵ2	0 (0)	0 (0)	
ϵ2/ϵ3	2 (1.8)	14 (13.2)	
ϵ2/ϵ4	0 (0)	2 (1.9)	
ϵ3/ϵ3	103 (92.8)	72 (68.0)	
ϵ3/ϵ4	5 (4.5)	14 (13.2)	
ϵ4/ϵ4	1 (0.9)	4 (3.7)	
Allele frequency			
ϵ2	2 (0.9)	16 (7.5)	
ϵ3	213 (95.9)	172 (81.1)	
ϵ4	7 (3)	24 (11.3)	
H-W-E *p*-value	0.104	0.114	
ApoE-ϵ4			
ϵ3/ϵ4, ϵ4/ϵ4	6 (5.4)	18 (16.9)	0.006^**^

χ^2^ test was performed. **p < 0.01.

### Association of the ApoE Genotype With EMT

In this study, we analyzed associations among the ApoE genotypes in the control and EMT groups. Interestingly, a higher number of E4 carriers (ϵ3/ϵ4, ϵ4/ϵ4) was found in the EMT group than in the control group (**Table 2**, *p* < 0.01).

### Hormone Levels in the Control and EMT Groups

The differences in hormone levels between the control group and the patients with EMT are shown in **Table 3**. No statistically significant differences in the levels of FSH (*p* = 0.798), LH (*p* = 0.930), or E2 (*p* = 0.172) were detected.

**Table 3:** FSH/LH/E2 levels in the control group and patients with EMT.

Parameters	Control (*n* = 111)	EMT (*n* = 106)	*t*/*z*	*p*-value
FSH	7.30 (6.27,8.55)	7.30 (5.74,8.88)	−0.256	0.798
LH	4.42 (3.27, 6.07)	4.75 (3.36, 5.83)	−0.088	0.930
E2	128 (59.5, 227.75)	156.5 (72.25, 238.30)	−1.365	0.172

Nonparametric test analysis was performed.

### Prediction Model for Patients With EMT

**Figure 2:**
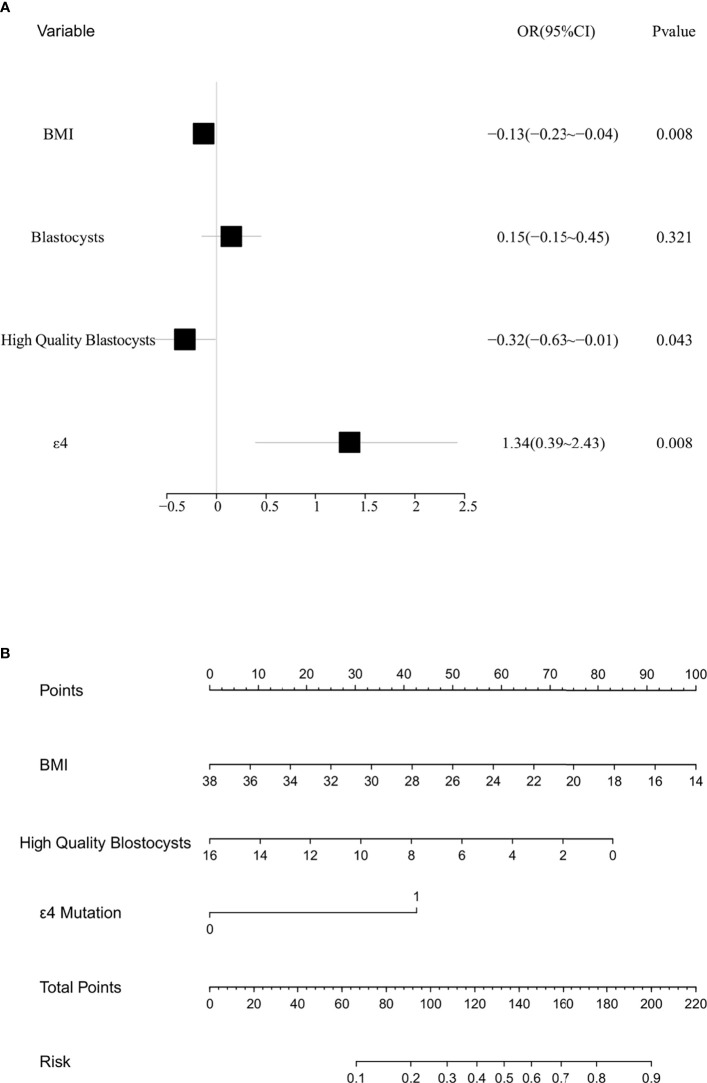
Multivariate analysis and risk assessment of the control subjects and patients with EMT. **(A)** Forest map from the multivariate analysis of the control subjects and patients with EMT; BMI, *p* = 0.008; high-quality blastocysts, *p* = 0.043; ApoE-ϵ4, *p* = 0.008. **(B)** Nomograph for predicting patients with EMT.

**Figure 3:**
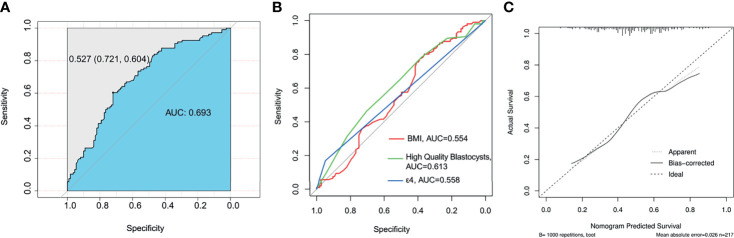
ROC curve of the prediction model and nomogram for patients suffering from EMT. The AUC value should be in the range of 0.5 to 1.0. An AUC of 0.5 corresponds to a random prediction, and the criterion 0.5 < AUC < 1 indicates that the model has predictive significance. **(A)** Multifactor sensitivity for predictingpatients with EMT; AUC=0.693. **(B)** Sensitivity to each of the following three factors for predicting patients with EMT: BMI, AUC=0.554; high-quality blastocysts, AUC=0.613; and ApoE-ε4, AUC=0.558. **(C)** A nomogram was drawn to assess the agreement between the predicted results and the observed actual results. The dashed lines represent the predicted results, and the solid lines represent the actual results. The close fit between the predicted results and the actual results indicates that the prediction effect could be considered good; mean absolute error = 0.026; *n* = 217.

As shown in **Figure 2A**, the results of the multivariate analysis showed that the BMI, high-quality blastocysts, and ApoE-ϵ4 were influencing factors for EMT. We then established a prediction model for patients suffering from EMT (**Figure 2B**). Receiver operating characteristic (ROC) analysis is usually used as a performance indicator to evaluate the quality of a prediction model. The area under the ROC curve (AUC) can be employed to assess the accuracy of the predictions made by the prediction model. To test the predictive ability of the established model for patients with EMT, the multifactor sensitivity (**Figure 3A**, AUC = 0.693) and each of the three factors sensitivity (**Figure 3B**, BMI, AUC = 0.554; high-quality blastocysts, AUC = 0.613; ApoE-ε4, AUC = 0.558) of the model for predicting EMT were investigated. We found that the multifactor prediction model exhibited better sensitivity than the single-factor prediction model. Subsequently, a correction curve was generated using the prediction model for patients suffering from EMT (**Figure 3C**).

## Discussion

In the present study, we investigated whether ApoE plays a vital role in the pathogenesis of EMT and found that ApoE expression was increased in the FF of patients with EMT compared with that of the control subjects. In addition, we analyzed the differences in the ApoE genotypes between the control and EMT groups. We found a higher number of E4 carriers (ϵ3/ϵ4, ϵ4/ϵ4) in the EMT group than in the control group (*p* < 0.01), which suggests an important role of ApoE-ϵ4 in the pathogenesis of EMT.

Previous studies have shown that ϵ3 is the most abundant ApoE genotype in the human population, with a frequency of 49%–90%, followed by ϵ4 with a frequency of 5%–37%, and ϵ2 was the least abundant genotype, with a frequency of 0%–14% (14). These findings are consistent with our analytical results that showed the proportion of ϵ3 was highest in both the control and EMT groups and that the proportion of ϵ2 in the control group was as low as 1.8%. Most studies on ApoE have focused on cardiovascular disease and Alzheimer’s disease risks due to its role related to lipids (21). However, the effects of ApoE go far beyond these diseases because this protein can affect a number of diseases, such as fertility, diabetes, and obesity (22–24).

ApoE is expressed in the endometrium and granulosa cells of the ovary to varying degrees (13). The involvement of lipoproteins and sterols in the regulation of ovarian function is complex due to the multitude of cell types and compartments in ovarian follicles. It has been reported that the growth of follicles is affected by the synthesis of hormones and growth factors (25), including ApoE, and the increase in ApoE observed in older women might be associated with the lower number of retrieved mature oocytes (18). Furthermore, an association between ApoE and spontaneous pregnancy loss might exist in patients with EMT (19), which suggests that ApoE plays a vital role in the pathogenesis of EMT. These findings are consistent with our results that the level of ApoE in the FF of patients with EMT was significantly higher than that in the FF of control subjects (*p* < 0.05).

This study revealed a difference in the BMI between the two groups (*p* = 0.024), which is consistent with the previous finding that patients with EMT have a lower BMI than control subjects (20). Although no significant difference in the number of eggs was found between the two groups (*p* = 0.091), we found that the numbers of blastocysts and high-quality blastocysts in the control group was significantly higher than that in patients with EMT. Blastocysts, particularly high-quality blastocysts, from patients with EMT were damaged to varying degrees. Furthermore, we also analyzed the hormone levels of the two groups and found no significant difference in the FSH, E2, and LH levels between the two groups, which is consistent with the results from previous studies (26). In addition, a multifactor prediction model for patients suffering from EMT was successfully established. This model exhibited good predictive significance, with an AUG value greater than 0.693. The established model also provides evidence that can be used to predict the occurrence and development of EMT.

This study has some limitations. First, previous studies have shown that inflammation is the central process in EMT, which can lead to pain, fibrosis, adhesion formation, and infertility (2, 27). Inflammation plays an important role in the etiology and pathophysiology of EMT (28, 29). Furthermore, ApoE-ϵ4 is reportedly associated with higher levels of inflammation (18). The cytokines in the human FF and the relationship between ApoE expression and the cytokines in the human FF of patients with EMT will be investigated in our future studies. In addition, an increasing number of studies have focused on the role of autophagy in EMT and have shown that autophagy plays a vital role in EMT (30). However, ApoE is reportedly associated with autophagy (31). As a result, ApoE might contribute to the development of EMT in part by regulating autophagy, which is a topic that we will investigate in the future. In addition, it has been reported that growth factors, such as BMP15 and GDF9, are local paracrine and autocrine factors that play an important role in regulating follicular development and ovarian function (32) and a significant role in the pathophysiology of EMT. The potential existence of an association between ApoE and BMP15 or GDF9 in the FF of patients with EMT is also a research topic that we will investigate in our future studies. Furthermore, the sample size should be increased in future studies.

In conclusion, this study provides further evidence showing that ApoE expression was increased in the FF and that ApoE4 is associated with EMT in Chinese women. In addition, we established a multifactor prediction model with good sensitivity for identifying patients who may suffer from EMT, and a combined analysis of three factors (BMI, high-quality blastocysts, and ϵ4) could be used to predict EMT. The effects of ApoE on the occurrence and development of EMT require further research.

## Data Availability Statement

The original contributions presented in the study are included in the article/supplementary material. Further inquiries can be directed to the corresponding authors.

## Ethics Statement

The studies involving human participants were reviewed and approved by the ethics committee of clinical medical research, First Affiliated Hospital of Anhui Medical University. The patients/participants provided their written informed consent to participate in this study.

## Author Contributions

Y-JL, FX, and KZ contributed equally to this work. Y-JL, Y-XC, XD, and J-PQ were responsible for the conceptualization and supervision of the study. FX and KZ performed the experiments, analyzed the data, and constructed the tables and figures. Y-JL, XD, and J-PQ designed the experiments and interpreted the data. M-YW, D-MJ, Y-HZ, Y-HX, AW, L-GS, S-MD, and Z-LW were responsible for technical and material support. FX wrote the original manuscript, which was edited by Y-JL, Y-XC, XD, and J-PQ. All authors read and approved the final version of the manuscript.

## Funding

The present work was supported by the National Natural Science Foundation of China (81771653), the Key Research and Development Program of Anhui Province (202004j07020043), the Nonprofit Central Research Institute Fund of the Chinese Academy of Medical Sciences (2019PT310002), the Key Science Research Project in Universities of Anhui Province (KJ2017A194), the Overseas Study and Research Program for Excellent Young Talents in Universities of Anhui Province (gxgwfx2021017), Research Fund of Anhui Institute of translational medicine (ZHYX2020A001), and the Key Project of Medical and Health Program of PLA (15DX009).

## Conflict of Interest

The authors declare that the research was conducted in the absence of any commercial or financial relationships that could be construed as a potential conflict of interest.

## Publisher’s Note

All claims expressed in this article are solely those of the authors and do not necessarily represent those of their affiliated organizations, or those of the publisher, the editors and the reviewers. Any product that may be evaluated in this article, or claim that may be made by its manufacturer, is not guaranteed or endorsed by the publisher.
